# Unbiased phage display screening identifies hidden malaria vaccine targets

**DOI:** 10.1080/22221751.2024.2429617

**Published:** 2024-11-12

**Authors:** Marcelo Jacobs-Lorena, Sung-Jae Cha

**Affiliations:** a Department of Molecular Microbiology and Immunology and Malaria Research Institute, Johns Hopkins Bloomberg School of Public Health, Baltimore, MD, USA; b Department of Medical Sciences, Mercer University School of Medicine, Macon, GA, USA

**Keywords:** Malaria, parasite ligand, host cell receptor, phage display, vaccine target

## Abstract

Malaria is among the deadliest infectious diseases. Over 200 million annual clinical malaria cases are reported and more than half a million people, mostly children, die every year. The most advanced RTS,S/AS01 vaccine based on the *P. falciparum* circumsporozoite protein (CSP), targets sporozoite liver infection but achieved modest efficacy. To reduce malaria death, novel malaria vaccine development is a high priority. Most malaria vaccine candidates target three infection steps: sporozoite liver infection, merozoite red blood cell (RBC) infection, and mosquito midgut infection. However, only few malaria vaccine candidates target specific parasite–host cell interactions. Our group has implemented the phage peptide-display approach to discover new parasite ligands and host cell receptors. Here we summarize our findings and discuss their potential for the development of novel vaccines.

## Introduction

Transmission of the malaria parasite starts when an infected mosquito deposits sporozoites in the skin of a vertebrate host [1,2]. Once in the blood stream, sporozoites home to, and infect the liver, where they develop within hepatocytes into hepatic schizonts [3–5]. Hepatic merozoites are released into the bloodstream to begin the asexual blood stage cycle by infecting RBCs [6]. A small proportion of the circulating parasites differentiate into male and female gametocytes. When ingested by a mosquito, gametocytes differentiate into gametes, followed by fertilization with the formation of zygotes and invasion of the midgut epithelium by the resulting motile ookinetes [7]. On the basal side of the midgut epithelium (facing the hemocoel) ookinetes differentiate into oocysts that when mature, each release thousands of sporozoites. These specifically target and invade the salivary glands, where they are kept until the mosquito bites the next vertebrate host, thus completing the transmission cycle.

There are two crucial bottlenecks in the malaria parasite life cycle, one at the infection of the liver and one in the mosquito, that constitute the principal malaria vaccine targets. The first bottleneck occurs at human infection that begins with the release into the skin of ∼50 *Plasmodium* sporozoites by an infected mosquito[1,2]. Some of these sporozoites enter the blood circulation and invade the liver in a process mediated by several interactions between parasite ligands and host cell receptors. First, the positively charged sporozoite CSP interacts with negatively charged glycosaminoglycans (GAGs) displayed in the lumen of the fenestrated sinusoids (liver blood vessels) by hepatic Stellate cells [7]. Next, sporozoites traverse the sinusoid lining, which is made up of endothelial cells and macrophage-like Kupffer cells [4,8,9]. Whereas few sporozoites succeed in infecting hepatocytes, if successful, each sporozoite generates around 3 × 10^4^ hepatic merozoites. These are released into the circulation and then expand geometrically by infecting RBCs, reaching up to a total of 10^12^ parasites that cause disease symptoms [1]. A vaccine that targets the liver stage bottleneck is referred to as pre-erythrocytic vaccine or PEV [10]. To date, only immunization targeting sporozoite liver infection has achieved sterile protection in humans [10,11]. The second bottleneck occurs at mosquito midgut infection [12,13]. When an infected blood meal is ingested by a female mosquito, *Plasmodium* gametogenesis is activated in the blood bolus. Gametocytes egress from host RBCs to transform into male and female gametes [13]. Each male gamete undergoes a drastic transformation known as exflagellation, leading to the formation of eight microgametes [14]. Microgametes actively move in the blood bolus in search of female gametes for fertilization, leading to the formation of zygotes. These further develop into motile ookinetes that actively move in the blood bolus until encountering and traversing the mosquito midgut epithelium. Movement then stops, and each ookinete located between the midgut epithelium and the basal lamina differentiates into an oocyst. This sequence of events is highly inefficient as out of thousands of gametocytes ingested only a few oocysts are formed [12]. A malaria vaccine that targets this mosquito stage bottleneck is referred to as transmission-blocking vaccine or TBV [10,15].

## The phage-display and mimotope approaches

A bacteriophage is a virus that infects bacteria. Phage display is a system that allows foreign peptides or proteins to be expressed on the surface of phage particles. The first application of the phage display technique in malaria research used the LX8 phage peptide-display library based on the M13 filamentous f88.4 phage [16,17]. Recombinant phages from this library display random 12-amino acid peptides, except for amino acids in positions 2 and 11 that are always cysteines. The cysteines form a disulfide bond resulting in an 8-amino acid loop. Importantly, and different from most phage libraries that display linear peptides, the LX8 library phages display circularized peptides that maintain a stable secondary structure. The library consists of an estimated 1.5 × 10^9^ different peptides. Because the recombinant 12-amino acid peptides on the surface of the phage particles are exposed to the environment, the library can be used to select displayed peptides that interact with any substrate.

**Figure 1.**
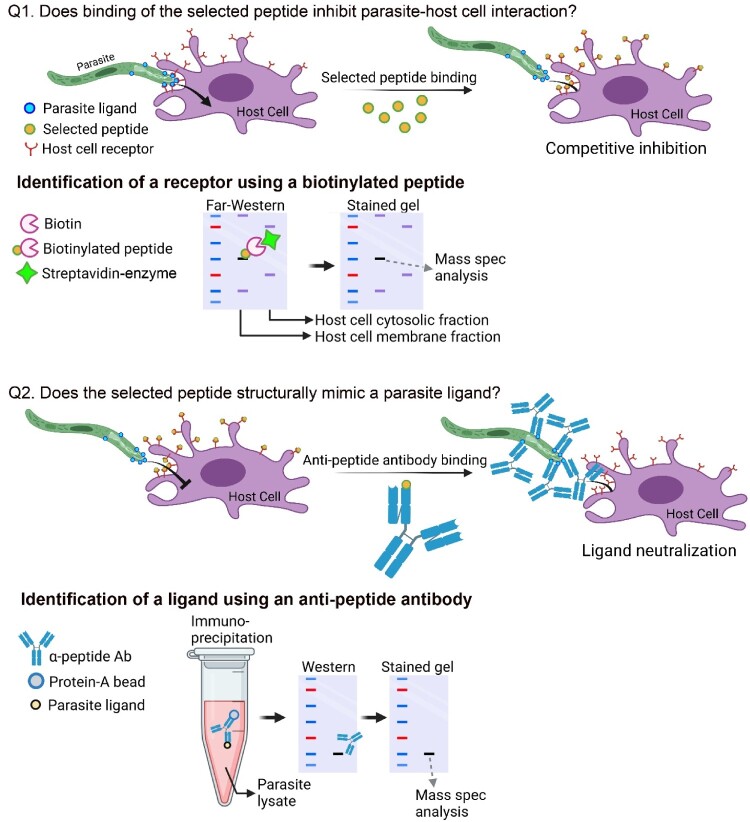
Two tests of the mimotope approach. After identification of a peptide that selectively binds to the host cell, the hypothesis that the peptide binds to a host cell parasite receptor can be tested with two functional assays: Q1. Does binding of the selected peptide inhibit parasite-host cell interaction? Inhibition of parasite-host cell interaction suggests that interference is mediated by a competition between the peptide and the parasite for binding to a host cell receptor. The host cell receptor can be identified by means of Far-Western blotting using the mimotope peptide as a probe. Host cells are fractionated into membrane and cytosolic fractions, the proteins are resolved by gel electrophoresis and transferred to a membrane which is then incubated with the biotinylated mimotope peptide. Peptide binding is then visualized using enzyme-conjugated streptavidin. Once the peptide-binding band is visualized (it should be in the membrane fraction), that position of the stained gel is cut for mass spectrometry analysis. Recombinant versions of the identified proteins are produced and the protein that is recognized by the peptide in an ELISA assay becomes the candidate receptor. An alternative to the Far-Western assay illustrated in the figure is to use the biotinylated peptide for a pull-down approach (not shown). Q2. Does the selected peptide structurally mimic a parasite ligand? The hypothesis being tested here is that the peptide mimics the conformation of a parasite surface protein domain, both of which (peptide and protein) bind to the same host cell receptor. That the anti-peptide antibody recognizes a specific parasite protein, and that this antibody inhibits parasite-host cell interaction, constitute evidence that the antibody recognizes a parasite ligand that interacts with the cell receptor. The host cell ligand can be pulled down from a parasite lysate with the anti-mimotope antibody in conjunction with Protein-A-conjugated agarose beads. The pulled down protein is fractionated by gel electrophoresis and position of the candidate ligand protein is confirmed using the same anti-mimotope antibody. The corresponding protein band of the stained gel is then cut for mass spectrometry. Recombinant protein(s) is/are then produced, and identity is confirmed with anti-mimotope antibody binding assays such as ELISA and/or Western blotting assays.

Our group has been using the LX8 library to target specific *Plasmodium* parasite–host cell interactions [16,18–25]. The working hypothesis is that to serve as a receptor, a protein should be relatively abundant and well exposed on the surface of the cell. This means that such receptors are readily accessible to the parasite, as well as to the peptides displayed by the phages. Repetitive screening for peptides that bind to a cell surface will enrich for peptides that bind to abundant cell surface molecules. A further consideration for the strategy of this approach is that the selected peptides play one of two possible roles: (1) the peptide binds to a host surface protein that serves as a receptor for the parasite, or (2) the peptide binds to a host surface protein that is irrelevant to parasite–host interaction. These two possibilities can readily be distinguished by testing whether or not the peptide competitively inhibits parasite–host cell interaction. In the first instance, it will inhibit interaction, and in the second, it will not. Perhaps surprisingly, every peptide that we have identified via screening of the phage library turned out to inhibit parasite–host interaction, thus identifying a putative receptor [16,18,19,22,23,26]. This aligns with the working hypothesis outlined above (Figure 1, Q1). The selected biotinylated peptide can then be used to molecularly identify the putative receptor, either by capturing the peptide together with the interacting protein(s) on a streptavidin column (“pull down”) or by using it as a probe on blots of host cell membrane proteins (Far-Western blotting), followed by identification by mass spectrometry. Identified recombinant protein(s) is/are then produced, and the one that interacts *in vitro* with the biotinylated peptide becomes the candidate receptor.

A second working hypothesis that we used is that the receptor-binding peptide mimics the molecular conformation of a parasite ligand epitope. In other words, the identified peptide is a mimotope of the parasite ligand, as both bind to the same receptor. This hypothesis can be tested by generating antibodies against the peptide and using Western blotting, ask whether the antibody recognizes a parasite molecule. Anti-peptide antibody binding to non-permeabilized parasites using an Immunofluorescence Assay (IFA) will verify that the protein identified by the antibody is on the surface of the parasite. An additional test is to show that this antibody prevents parasite–host cell interaction by binding to the putative parasite ligand. Perhaps also surprising, in every case the anti-peptide antibody led to the identification of a putative parasite ligand (Figure 1, Q2).

Once a candidate ligand and receptor are identified, a final test is to show that the two recombinant proteins interact. This test is usually performed by showing that one of the proteins immobilized on a solid substrate binds the other protein in solution.

Significantly, phage display screening is an unbiased approach that targets parasite–host cell interaction without any a priori knowledge of properties of the interacting partner proteins.

## The liver bottleneck

**Figure 2.**
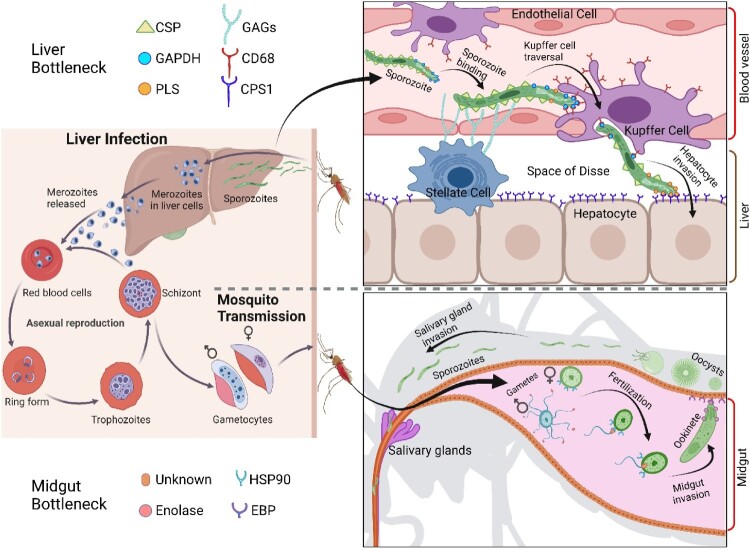
Identification of [parasite ligand]-[host cell receptor] pairs at parasite population bottlenecks. Upper panels. The parasite bottleneck of vertebrate infection occurs at liver infection. Circulating sporozoites bind to the liver endothelium via interaction between sporozoite CSP and Stellate cell GAGs. Arrested sporozoites traverse the liver endothelial lining utilizing the phagocytic Kupffer cell machinery. The Kupffer cell-binding peptide P39 inhibits Kupffer cell-sporozoite interaction. Using P39, CD68 was identified as a Kupffer cell receptor and surface GAPDH was identified as the sporozoite ligand (see Figure 1). After traversing the liver endothelial lining, sporozoites selectively infect hepatocytes. The hepatocyte-binding peptide HP1 inhibits hepatocyte-sporozoite interaction. Using HP1, carbamoyl-phosphate synthase (CPS1) was identified as a hepatocyte receptor and surface phospholipid scramblase (PLSCR) was identified as the sporozoite ligand. Lower panels. The parasite bottleneck at mosquito infection occurs in the midgut. Asexual-stage parasites do not survive in the blood bolus whereas gametocytes rapidly form male and female gametes, followed by fertilization and differentiation into motile ookinetes that cross the mosquito midgut epithelium. Identification of the mimotope MG1 peptide that binds to male gametes led to the identification of the female surface HSP90 fertilization ligand. The male fertilization receptor has not yet been identified. Identification of the mosquito midgut mimotope SM1 peptide led to the discovery of the ookinete surface enolase ligand and of the EBP midgut epithelium receptor.

The liver is the initial site of *Plasmodium* replication in the vertebrate host, where parasites transform into merozoites that ultimately infect RBCs and cause disease symptoms. Although few of the sporozoites deposited in the skin by an infected mosquito reach the hepatocytes, one successful infection is sufficient to generate more than twenty thousand hepatic merozoites [6]. The most advanced malaria vaccine targets this liver bottleneck [10]. However, sporozoites circulating in the blood stream have no direct contact with their first infection target, the hepatocytes. To reach the liver parenchyma, circulating sporozoites must first recognize when they have reached the liver. The liver has specialized fenestrated blood vessels called sinusoids that are lined by two main cell types: endothelial cells similar to cells that line all blood vessels and Kupffer cells, that are liver macrophages (Figure 2). The Space of Disse is the space between the sinusoidal wall and the liver parenchyma. Within the Space of Disse resides a third cell type – the stellate cells – whose function includes fat storage and secretion of an extracellular matrix [27]. Recognition of the liver by circulating sporozoites is mediated by the binding of sporozoite surface proteins (CSP and thrombospondin-related adhesive protein or TRAP) to the liver-specific and highly sulfated glycosaminoglycans (GAGs) [28]. These GAGs are synthesized by the stellate cells in the space of Disse and protrude into the sinusoidal lumen through sinusoid fenestrae (openings) [7,29,30]. The diameter of the sinusoidal fenestrae is about 1/10 of the sporozoite diameter and therefore, are too small for sporozoites to pass through. Thus, to reach the space of Disse, sporozoites must traverse the endothelial or Kupffer cells of the sinusoidal wall.

### Exit the circulation

*Plasmodium* sporozoites preferentially traverse Kupffer cells by transcytosis [3,4], but may also penetrate endothelial cells [9]. Sporozoite-Kupffer cell interaction was investigated using phage display screening. A Kupffer cell-binding peptide – P39 – was identified by screening the LX8 library for phages that bind to rat primary Kupffer cells in culture [21]. A recombinant phage that displays the P39 peptide bound to the Kupffer cell surface and strongly inhibited sporozoite-Kupffer cell interaction in culture. Moreover, intravenous injection of the P39-displaying phage strongly reduced parasite liver load in mice. To rule out possible steric hindrance by the recombinant phages, a synthetic biotinylated P39 peptide was generated. This peptide bound to the Kupffer cell surface and inhibited both *P. berghei* and *P. falciparum* sporozoite-Kupffer interaction, confirming that inhibition is not due to steric hindrance. To identify the target of the P39 peptide on the Kupffer cell surface, a primary rat Kupffer cell membrane fraction was resolved by SDS-PAGE and transferred onto a PVDF membrane which was further incubated with biotinylated P39 peptide. A single band, detected with alkaline phosphatase-conjugated streptavidin, was eluted and identified as CD68 by mass spectrometry (Figure 1, Q1). Hypothesizing that P39 is a mimotope of a sporozoite ligand for Kupffer cell interaction, it was shown that anti-P39 antibodies bind to *P. berghei* and *P. falciparum* sporozoites and strongly inhibit *in vitro* sporozoite-Kupffer interaction [22]. Importantly, immunization with the P39 peptide strongly protected mice from infection when bitten by infected mosquitoes. Immunoprecipitation with an anti-P39 antibody and mass spectrometry identified glyceraldehyde 3-phosphate dehydrogenase (GAPDH) as the putative ligand on the sporozoite surface (Figure 1, Q2). Which domain of the GAPDH protein is recognized by the anti-P39 antibody? To answer this query, Western blotting assays of GAPDH protein fragments determined that the anti-P39 antibody recognizes a domain within the C’-terminal-end 65 amino acids. Using an overlapping 15-amino-acid-long peptide library covering these 65 amino acids, two short epitopes were identified. Significantly, antibodies against each of these two epitopes inhibited sporozoite mouse liver infection with similar efficacy as the anti-P39 antibody itself [31].

### Hepatocyte infection

After traversing the liver sinusoidal lining, *Plasmodium* sporozoites specifically infect hepatocytes. As this is the strongest parasite population bottleneck, it is a prime target for the development of a PEV. Screening the LX8 library for phages that bind to mouse primary hepatocytes led to the identification of the HP1 hepatocyte-binding phage [23]. This recombinant phage bound to hepatocytes and inhibited sporozoite-hepatocyte interaction. Antibodies against the HP1 recombinant phage bound to *P. berghei* sporozoites and inhibited hepatocyte interaction *in vitro*. Further experiments used a synthetic HP1 peptide to exclude the possibility of steric hindrance by the phage. The biotinylated HP1 peptide selectively bound to hepatocytes, not to macrophages. An anti-HP1 antibody bound to *P. berghei* and *P. falciparum* sporozoites and by doing so, inhibited sporozoite-hepatocyte interaction. Western blotting assays with an anti-HP1 antibody identified a ∼50 kDa *P. berghei* sporozoite surface protein, further identified by mass spectrometry as the phospholipid scramblase (PLSCR), a potential vaccine antigen. Indeed, antibodies against PbPLSCR strongly inhibited *P. berghei*- and *P. falciparum*-sporozoite interactions with hepatocytes. Far-Western blotting assay identified a 150 kDa hepatocyte membrane protein as the target of HP1 peptide binding and a putative sporozoite receptor. Pull-down and mass spectrometry assays identified it as carbamoyl-phosphate synthase (CPS1). Direct interaction between sporozoite PbPLSCR and hepatocyte CPS1 was confirmed with pull-down and ELISA assays.

## The mosquito bottleneck

A strong bottleneck occurs as the *Plasmodium* parasite develops in the mosquito midgut (Figure 2) and this stage is a prime target for TBV [10,12,13,15]. Once infected blood is ingested by a female mosquito, mature gametocytes are induced by environmental and mosquito factors such as decreasing temperature, pH increase, and mosquito xanthurenic acid to develop into gametes [32]. The first cellular interaction in the mosquito occurs between male and female gametes at fertilization. The second cellular interaction occurs at ookinete traversal of the midgut epithelium.

### Fertilization

The most advanced TBV vaccine antigen is P230, a protein that plays an important role in male gamete development [10]. Other TBV candidates, such as P48/45 and P47 are essential proteins for gamete development. To date, no TBV antigen that targets fertilization has been reported. To identify a fertilization ligand and receptor pair the LX8 library was screened separately against isolated female and male *P. berghei* gametes leading to the identification of the female gamete-binding FG1 and the male gamete-binding MG1 recombinant phages [24,25]. Assays with synthetic peptides confirmed that the inhibition by recombinant phages were not due to steric hindrance. Binding of the MG1 peptides to *P. berghei* male gametes inhibited fertilization *in vitro.* Importantly, both the MG1 peptide and anti-MG1 antibodies inhibited fertilization when both *P. berghei* and *P. falciparum* where administered to mosquitoes. To test the hypothesis that MG1 is a mimotope of a putative female ligand for male gamete interaction (Figure 1, Q2), Western blotting determined that an anti-MG1 antibody recognizes a ∼110 kDa female gamete membrane protein, further identified as *Plasmodium* heat-shock protein 90 (HSP90) [25]. Significantly, HSP90 has been reported to be a vertebrate gamete membrane protein essential for fertilization [33–35].

### Ookinete midgut invasion

*Plasmodium* ookinetes cross the mosquito midgut epithelium using diverse proteins [12,36]. For targeting this specific interaction, LX8 library phages were fed to *Anopheles gambiae* female mosquitoes, thus putting the recombinant phages in direct contact with the epithelial cells that are invaded by the ookinetes. This screen enriched for the SM1 recombinant phage; it specifically bound to the luminal side of the midgut epithelium, but not to the midgut surface facing the hemocoel and did not bind to ovaries [16]. SM1 phage binding dramatically inhibited *P. berghei* ookinete mosquito midgut invasion. Binding and inhibition assays using SM1 phages were repeated with biotinylated SM1 peptide, thus excluding the possibility of steric hindrance by phage particles. Importantly, incorporation of the SM1 peptide into a *P. berghei­*-infected blood meal strongly inhibited ookinete midgut invasion. These findings led to the engineering of a mosquito that secretes the SM1 peptide into the gut upon blood feeding [37]. This was the first report of a transgenic mosquito impaired for transmission of the malaria parasite. Further study determined that an anti-SM1 antibody binds to a *Plasmodium* ookinete surface enolase, a putative ligand for interaction with the mosquito midgut [18]. In support of this hypothesis, it was found that anti-Pf-enolase antibodies strongly inhibit *P. berghei* and *P. falciparum* ookinete invasion of the midgut. The mosquito midgut protein bound by SM1 (putative receptor) was identified as Enolase Binding Protein (EBP) [20]. Of note, in addition to interacting with the mosquito midgut epithelium, ookinete enolase also binds plasminogen [18], which facilitates ookinete movement in the mosquito blood bolus and sporozoite infection of the mammalian host [38,39].

## Other applications

Besides the ligand–receptor pair identification, phage peptide display has been widely used for drug discovery and vaccine development. For drug discovery, phage peptide screening has been applied for selecting functional peptides since binding of a peptide to a protein can alter the target molecule conformation. For examples, membrane receptor binding peptides have been selected using phage peptide display library screening, which were further tested for antagonistic or agonistic activities in receptor-mediated signal transduction pathways [40]. Peptides have also been selected for production of an antidote of which binding neutralizes its toxicity [41]. For vaccine development, peptides have been screened as epitope mimics of monoclonal antibodies targeting carbohydrates or hydrophobic moieties, as these molecules cannot be easily synthesized [42–45]. Such selected mimotope peptides can be used for the generation of protective vaccines.

## Concluding remarks

Unbiased screening using a phage peptide-display library led to the identification of mimotope peptides that specifically target sporozoite-Kupffer cell interaction, sporozoite-hepatocyte interaction, gamete fertilization, and ookinete-midgut invasion (Figure 2). Using antibodies against the identified mimotope peptides, sporozoite surface GAPDH, sporozoite surface PLSCR, female gamete surface HSP90, and ookinete surface enolase were identified as parasite ligands for liver entry, hepatocyte infection, gamete fertilization, and mosquito midgut invasion, respectively (Figure 2). These newly identified parasite proteins had never been investigated as *Plasmodium* ligands. These are abundant parasite proteins that are involved in essential housekeeping pathways and perform moonlighting functions as surface ligands. The high conservation of these moonlighting proteins with their host counterparts has been hypothesized to protect the parasites, as the host would not elicit antibodies against these conserved proteins [46]. However, parasite HSP90, GAPDH and enolase have been investigated as vaccine candidates of diverse infectious diseases, as these proteins have regions that evolutionarily diverged [47–53]. Notably, all identified mimotope peptides elicited protective or transmission-blocking immunity, implying that the mimotope peptides themselves could be used as vaccine antigens. Taking advantage of their small sizes, these peptides could be fused to other antigens, thus broadening vaccine targets while avoiding generation of non-protective antibodies and improving protection efficacy [53].

An important property of the LX8 library is that the displayed peptides have a secondary structure conferred by the disulfide bond between the cysteines in positions 2 and 11. For instance, when this bond was disrupted either by reducing agents or mutation to alanine, the SM1 peptide did not bind anymore to the mosquito midgut epithelium (unpublished results).

Finally, we note that the phage display approach is not restricted to investigation of the malaria parasite cycle, but can be applied to investigate the interaction between any two cell types [54–56].
